# Idiopathic Granulomatous Appendicitis: A Case Report and Review of the Literature

**DOI:** 10.7759/cureus.82081

**Published:** 2025-04-11

**Authors:** Abanoub Awad, Isaac Theerman, Jason Beckermann

**Affiliations:** 1 Surgery, Mayo Clinic Health System, Eau Claire, USA

**Keywords:** acute appendicitis, appendicitis, appendicular mass, granulomatous appendicitis, idiopathic granulomatous appendicitis

## Abstract

Idiopathic granulomatous appendicitis (IGA) is a rare condition that clinically presents similar to acute appendicitis, with symptoms such as abdominal pain, fever, and anorexia. We report a case of a 30-year-old postpartum female who presented with symptoms of acute appendicitis. Imaging revealed a thickened appendix, and laparoscopic appendectomy was performed. Intraoperative findings led to conversion to laparotomy with ileocolic resection. Histopathology confirmed granulomatous appendicitis with focal suppuration and no evidence of Crohn's disease or infection. IGA is diagnosed by excluding other infectious or non-infectious causes, and appendectomy is the treatment of choice, with a favorable prognosis. The condition requires careful pathological evaluation to avoid unnecessary extensive surgery.

## Introduction

Acute appendicitis is the most common cause of acute abdominal pain and the most common surgical emergency worldwide [1, 2]. Historically, open appendectomy was the standard surgical approach; however, laparoscopic appendectomy is now the standard of care in the United States, as it's associated with less post-operative pain and a faster recovery [1, 3]. Granulomatous appendicitis (GA) is not a well-understood phenomenon, mostly due to its rare occurrence in practice; it's reported only in 0.1% to 2% of all appendectomies [4].

GA is either primary or secondary to infectious agents like *Mycobacterium tuberculosis* (TB), *Yersinia*, and parasitic infestation [5, 6, 7] or non-infectious causes like sarcoidosis and Crohn's disease [6, 8, 9]. 

Written informed consent was obtained from the patient for the publication of her protected health information as a case report. Informed consent is available upon request.

## Case presentation

We present a case of a 30-year-old, 12 weeks postpartum female, without significant past medical history, who presented to the emergency room with one day of anorexia, chills, fever, and abdominal pain that started diffusely but then was localized to the right iliac fossa.

**Figure 1:**
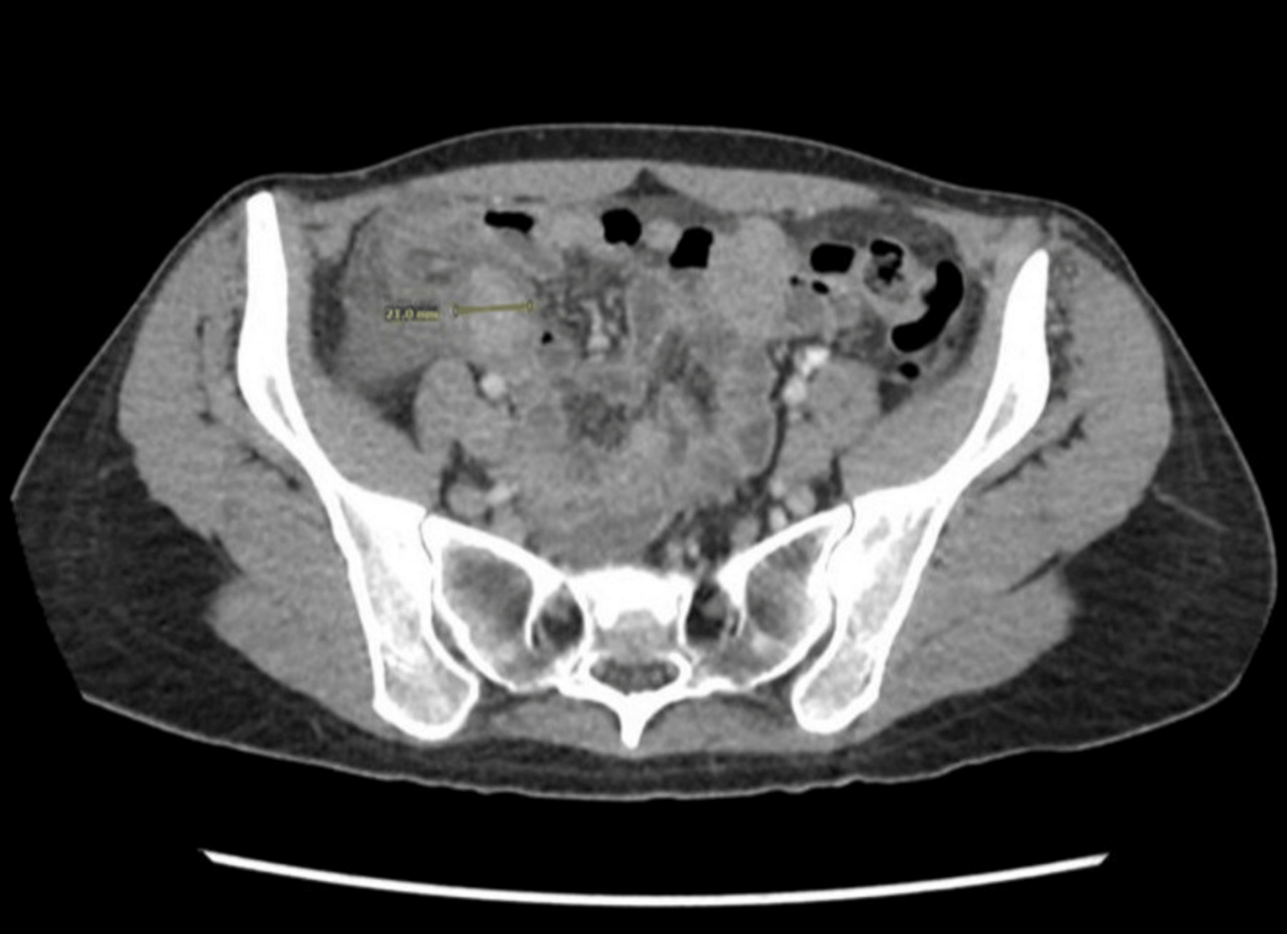
Axial section showing thickened appendix with surrounding phlegmon and edema

**Figure 2:**
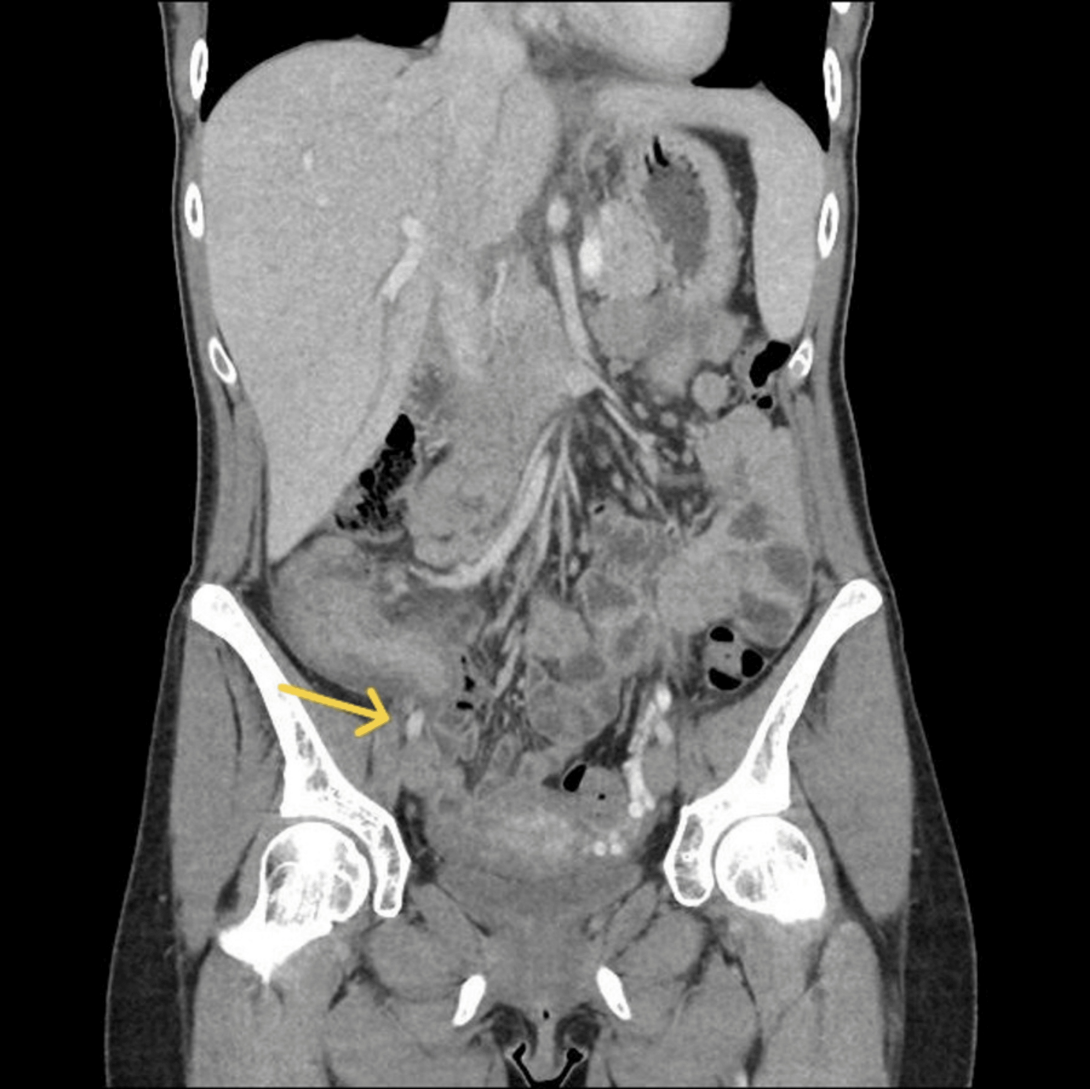
Coronal section showing thickened appendix (arrow)

By abdominal examination, there was right iliac fossa tenderness with decreased abdominal sounds; the patient was clinically diagnosed with acute appendicitis. The patient's total leucocytic count was 7.6 × 10³/µL (reference range: 4.0-11.0 × 10³/µL), B-hCG was negative, and she had unremarkable urinalysis. A computed tomography (CT) scan was done, revealing a thickened appendix (18 mm) with surrounding phlegmon, edema, and a small amount of free fluid in the pelvis (Figures 1, 2)

**Figure 3:**
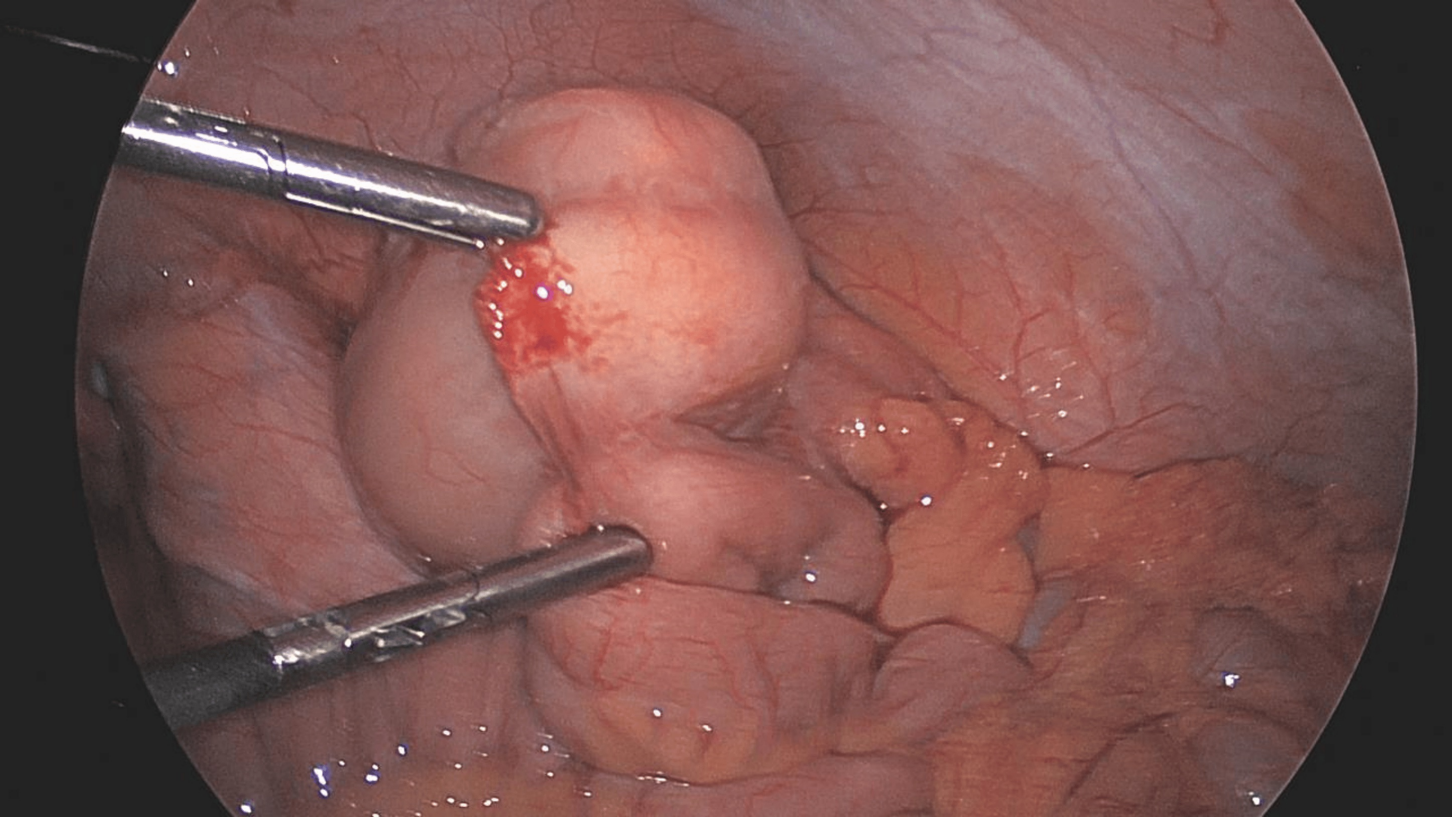
Intra-operative finding of an appendicular mass, which appeared suspicious for a neoplastic process, leading to conversion to laparotomy for further assessment

The patient was taken to the operating theatre for a routine laparoscopic appendectomy. After gaining visual access to the abdomen, there was noted to be an inflamed appendix along with what appeared to be a neoplastic process, as the cecum was noted to be thickened. A biopsy of mucinous material in the anterior abdominal wall was sent to pathology and noted to be inflammatory in nature; however, the procedure converted to laparotomy for better visualization and assessment (Figure 3).

**Figure 4:**
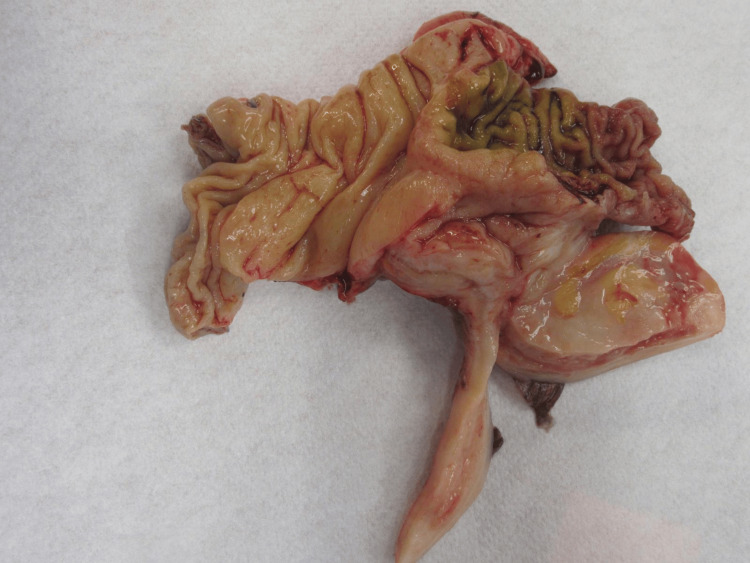
Specimen after resection, showing the appendix along with the surrounding tissue removed during the ileocolic resection

After further evaluation, there were no additional sites of mucin, no studding, or carcinomatosis, and the decision was to perform ileocolic resection with anastomosis. However, many lymph nodes appeared prominent, and close attention was paid to the surgical margins along with mesenteric tissue resected to ensure that an adequate margin and number of lymph nodes were captured in the specimen for cancer staging (Figure 4).

Surgery was well tolerated; the post-operative course was uneventful, with the patient being discharged on post-operative day three.

**Figure 5:**
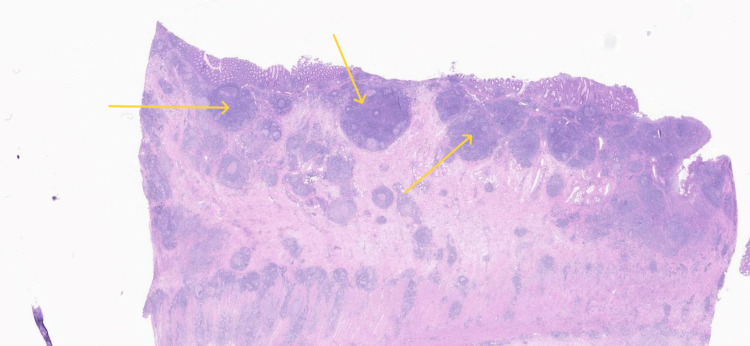
Histopathology showing granuloma formation under hematoxylin and eosin (H&E) staining, indicating chronic inflammation with granulomatous changes in the appendix

**Figure 6:**
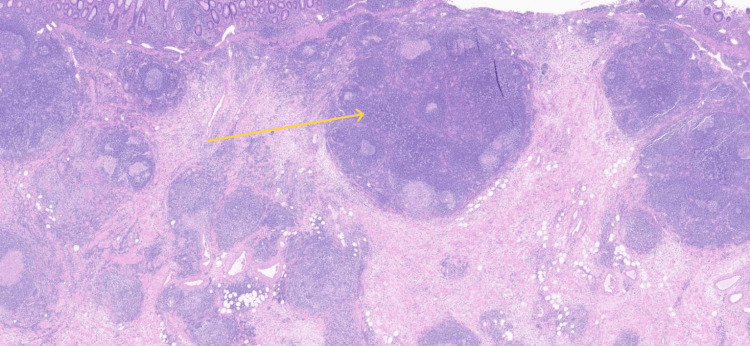
Histopathology showing granuloma formation with multinucleated giant cells in the wall of the appendix

Surgical pathology was consistent with granulomatous appendicitis with focal suppuration. The mucosa showed active chronic inflammation with scattered crypt abscesses and mild crypt architectural distortion. There were underlying transmural lymphoid aggregates associated with extensive granulomatous inflammation and peri-appendiceal fibrosis (Figures 5, 6). Twenty-two lymph nodes were negative for malignancy. The differential diagnosis included infection (*Yersinia*, fungal, mycobacterial), Crohn's disease, and idiopathic granulomatous appendicitis. 

Three months later, the patient came for a colonoscopy to exclude inflammatory bowel disease or other underlying pathologies; there was no endoscopic evidence of any abnormality in the entire colon. Multiple random biopsies were obtained, revealing normal colonic mucosa.

## Discussion

Granulomatous appendicitis is a type of chronic non-specific inflammation in the appendix that results in the formation of a granuloma, often as a response to infectious or other non-infectious agents [10]. It's very rare to encounter GA in clinical practice [4]. 

A few recent reports describe idiopathic granulomatous appendicitis as a new disease entity of isolated granulomatous inflammation of the appendix [11, 12].

Historically, it was believed that GA is an isolated form of Crohn's disease, but now it's believed that there are many potential causes of it, either idiopathic (primary) or secondary due to infectious or non-infectious causes [4, 13].

Distinguishing between idiopathic granulomatous appendicitis and isolated appendiceal Crohn's disease is difficult in the perioperative period; however, the prognosis of idiopathic granulomatous appendicitis is more favorable [14]. 

Although it's now believed that GA could occur as an isolated process, either idiopathic or Crohn's related, it remains unrelated to Crohn's disease or its future development. However, the presence of GA in an appendectomy specimen should prompt a search for Crohn's disease elsewhere in the bowel [12, 15]. Idiopathic granulomatous appendicitis is a lesion of exclusion, meaning that infectious and non-infectious causes must be excluded first prior to diagnosis [12]. 

During the surgery, the appendix appears with a firm consistency, thick walls, and with increased blood supply, it may resemble a neoplasm, which can lead to concerns about malignancy [16]. 

Treatment of granulomatous appendicitis is appendectomy [11], However, macroscopic features found intra-operatively may sway surgeons to perform an open approach with a more extensive resection, due to concerns of malignancy once final histopathology is completed [16], therefore, microscopic examination is important in intra-operative diagnosis of this lesion to prevent any unnecessary surgical procedures [17]. Microscopic examination was not performed in this case as there was noted to be sufficient tissue and lymph nodes in the specimen for both diagnostic and staging purposes. 

## Conclusions

Idiopathic granulomatous appendicitis is a rare condition that closely mimics acute appendicitis but is characterized by granulomatous inflammation. Careful diagnostic evaluation to exclude both infectious and non-infectious causes is recommended.

Treatment typically involves appendectomy, and the prognosis is generally favorable. Proper pathological evaluation is crucial to avoid unnecessary extensive surgical procedures and ensure an accurate diagnosis.
